# Clinical research activities during COVID-19: the point of view of a promoter of academic clinical trials

**DOI:** 10.1186/s12874-021-01291-0

**Published:** 2021-04-30

**Authors:** Linda Valmorri, Bernadette Vertogen, Chiara Zingaretti, Anna Miserocchi, Roberta Volpi, Alberto Clemente, Isabella Bondi, Irene Valli, Britt Rudnas, Giovanni Martinelli, Oriana Nanni

**Affiliations:** 1 Unit of Biostatistics and Clinical Trials, Istituto Scientifico Romagnolo per lo Studio e la Cura dei Tumori (IRST) IRCCS, Via P. Maroncelli 40, 47014 Meldola, Italy; 2 Istituto Scientifico Romagnolo per lo Studio e la Cura dei Tumori (IRST) IRCCS, Meldola, Italy

**Keywords:** COVID-19, Promoter, No profit, Management, Clinical trials

## Abstract

**Background:**

During the COVID-19 emergency, IRST IRCCS, an Italian cancer research institute and promoter of no profit clinical studies, adapted its activities and procedures as per European and national guidelines to maintain a high standard of clinical trials, uphold participant safety and guarantee the robustness and reliability of the data collected. This study presents the measures adopted by our institute with the aim of providing information that could be useful to other academic centers promoting clinical trials during the pandemic.

**Main text:**

After an in-depth analysis of European and Italian guidelines and consultation and analysis of publications regarding the actions implemented by international no profit clinical trial promoters during the emergency, we monitored the way in which the institute managed clinical trials, verifying compliance with regulatory guidelines and clinical procedures, and evaluating screening and recruitment trends in studies. During the pandemic, our center activated a new clinical trial for the treatment of patients with COVID-19. A number of procedural changes in clinical trials were also authorized through notified amendments, in accordance with Italian Medicines Agency (AIFA) guidelines. Patient screening and enrolment was not interrupted in any site participating in multicenter interventional clinical trials on drugs. The institute provided clear indications about essential procedures to be followed, identifying those that could be postponed or carried out by telephone/teleconference. All external sites were monitored remotely, avoiding on-site visits. Although home-working was encouraged, the presence of staff in the central office was also guaranteed to ensure the continuity of promoter activities.

**Conclusions:**

Some measures adopted by IRST could also be effective outside of the COVID-19 period, e.g. numerous activities relating to clinical trial management could be performed on a home-working basis, using suitable digital technologies. In the future, electronic medical records and shared guidelines will be essential for the correct identification and management of trial risks, including the protection of the rights and privacy of subjects taking part. Promoter supervision could be increased by implementing centralized monitoring tools to guarantee data quality. Closer collaboration between promoters and local study staff is needed to optimize trial management.

**Supplementary Information:**

The online version contains supplementary material available at 10.1186/s12874-021-01291-0.

## Background

Italian legislation no. 23, art. 40, of April 8, 2020 established urgent regulations for pharmacological clinical trials during the COronaVIrus Disease 19 (COVID-19) emergency. The Italian Medicines Agency (Agenzia Italiana del Farmaco - AIFA), the Italian competent authority for pharmacological clinical trial evaluation and, in particular, for studies on patients with COVID-19 (legislation no. 18, art.17 of March 17, 2020, entitled “Cura Italia”), published several press releases on the matter. The most recent was that of April 7, 2020, which provided indications for the management of clinical trials and of substantial trial amendments following the exceptional restrictive measures introduced by the Italian government to cope with the pandemic. The measures adopted were to be valid until further notice and were closely linked to the state of emergency approved by the Council of Ministers on January 31, 2020.

On April 28, 2020, the European Medicines Agency (EMA) published specific guidelines [1] establishing simplification measures to be implemented during the public health crisis until their revocation by the European Union/European Economic Agreement (EU/EEA). European and Italian guidelines have promoted pragmatic and harmonized actions capable of guaranteeing the flexibility and procedural simplifications needed to maintain the integrity of clinical trials, the rights, safety and wellbeing of trial participants, and the safety of clinical trial staff. The main recommendations of EMA and AIFA are shown in Table 1.

**Table 1:** Main recommendations of EMA and AIFA during the COVID-19 pandemic

	EMA (28/04/2020)	AIFA (07/04/2020)
Changes to ongoing trials, safety reporting and risk assessment Safety reporting Risk assessment	Sponsors should include in their risk assessment the most appropriate measures needed during COVID-19: • Conversion of physical visits to phone or video visits, postponement or complete cancellation of visits. • Interruption or slowing down of recruitment of new trial participants; • Extension of trial duration; • Transfer of trial participants to investigational sites away from risk zones, or closer to their home, to sites already participating in the trial, or new ones. • Acceptable to perform laboratory, imaging or other diagnostic tests (e.g. blood cell count, liver function test, X-ray, CT, MRI, ultrasonography, ECG etc.), at a local laboratory or relevant clinical facility authorised/certified. These analyses can be used for safety decisions. Sponsors must continue safety reporting. Investigators must continue collecting adverse events through alternative means, e.g. by phone or telemedicine visits.	Promoters must define risk assessment plans and implement suitable actions. An accurate tracking of all deviations from study procedures and the rapid implementation of emergency measures must be ensured through notified amendments: • Temporary suspension of enrolment and/or treatment or change in trial procedures to limit visits to those absolutely needed (if possible, also providing for an extension of trial duration). • Specific procedures can be performed directly at the patient’s home by experimental center staff or by third parties; these may include both clinical procedures (e.g. registration of adverse events, vital signs, etc.), and the administration of non-self-administering therapies (e.g. infusions). Compliance with personal data protection rules remains applicable, such as the need for investigator’s supervision and maintenance of efficient communication between personnel in charge, investigator and patient. • Laboratory and/or instrumental examinations (e.g. CT, MRI, X-ray) can be performed at facilities close to the subject’s home.
Communication with authorities	Priority is given to any (new) clinical trial application for the treatment or prevention of COVID-19, and/or substantial amendment applications to existing clinical trials necessary as a result of COVID-19.	Preliminary evaluation of COVID-19 treatment studies will be carried out by AIFA Scientific Technical Commission (CTS). After CTS approval, trials will be evaluated by the AIFA competent authority (Clinical Experimentation Office) and by the Ethics Committee of the National Institute for Infectious Diseases Lazzaro Spallanzani (INMI) in Rome (appointed as the National Ethics Committee). An accelerated time-frame is expected for the evaluation of COVID-19 studies.
Agreement with and communication between sponsors, trial sites and trial participants	Changes to trial procedures should be clearly communicated by the Sponsor and agreed upon by the investigators.	Suitable remote communication mechanisms between the interested parties must be provided. It is recommended that adequate documentation about all communications be maintained.
Changes to informed consent	Alternative procedures to obtain informed consent: phone or video-calls to obtain oral consent, to be documented in trial participants’ medical records, supplemented with e-mail confirmation. Any validated and secure electronic system already used in the trial for obtaining informed consent can be used as per usual practice if in compliance with national legislation.	Alternative procedures to obtain informed consent must be considered: telephone contact, followed by confirmation e-mail or validated electronic system (previous DPO authorization). Written consent must be obtained as soon as the situation permits. DPO must identify technical and organizational measures to prove the acquisition of a valid consent.
Changes in the distribution of the investigational medicinal products	Changes in the distribution of IMP may be necessary to prevent avoidable visits to sites and to provide trial participants with needed treatments. Sponsors must assess the risks relating to the product and consider any alternative shipping and storage arrangements. The continuation of treatment should be under adequate supervision of the investigator.	AIFA allows the experimental center to supply, during patient visit to the center, a drug quantity that covers a longer period of time than that normally estimated by protocol and with an expiration date that goes beyond the treatment period provided in order to avoid the risk of patients taking an expired drug. Drug delivery to a family member or to a delegated caregiver is also permitted if the patient has difficulty in reaching the experimental center. The clinician must constantly ensure that the drugs are taken correctly. Drug delivery from the hospital Pharmacy directly to the patient’s house is allowed through dedicated couriers. The Pharmacy of the experimental center must supervise the process and inform the investigator as per correct trial management and according to the risk plan provided by the sponsor, considering type of IMP, method of distribution, conservation and transport. Documentation related to IMP delivery must be stored at the experimental site to guarantee the confidentiality of the data.
Changes to monitoring	A risk-based approach to monitoring should be taken, focusing on certain sites, data points and processes that are critical to ensure the rights, safety and well-being of trial participants and the integrity of the trial (and trial data). a) On-site monitoring. The cancellation or postponement of on-site monitoring visits and the extension of the period between monitoring visits are likely to be necessary. b) Centralised monitoring and central review of data collected can supplement and temporarily replace on-site monitoring. c) Off-site monitoring including phone calls, video visits, e-mails or other online tools can be used to discuss trial progress and participant status with the investigator and site staff, to resolve problems, review procedures and facilitate remote site selection and investigator training. d) Remote source data verification (SDV) will currently only be considered necessary for very few trials when in line with national law. It is essential that robust follow-up measures are planned and can be rapidly implemented when the situation normalizes.	Sponsors must define risk assessment plans and implement actions that take into account the need to reduce unnecessary contact during the COVID-19 period, evaluating whether on-site monitoring visits can be deferred or replaced by the introduction and/or strengthening of the centralized monitoring. Exceptional and alternative methods are accepted for SDV, such as telephone contact or, better, video conferences with the experimental site staff. Remote monitoring methods may be taken into consideration when appropriately justified by the intent to protect the rights and well-being of the subjects enrolled in the trial. It is essential for promoters to plan robust follow-up measures to assess and eventually fill in the gaps due to the reduced frequency of in-site monitoring or to the use of alternative measures.
Reimbursement of exceptional expenses	Urgent safety measures may create unplanned expenses that should be borne by the sponsor, preferably directly. If these expenses have to be borne initially by the trial participants, these should typically be compensated subsequently by the sponsor via the investigator.	AIFA recommends that the promoter reimburses documented costs incurred by patients for the implementation of urgent measures.

Istituto Scientifico Romagnolo per lo Studio e la Cura dei Tumori (IRST) IRCCS is an Italian institute for cancer research and treatment. The center is part of the Emilia-Romagna Regional Health Service and represents the hub of the Cancer Network of Romagna. In 2012, IRST was formally recognized by the Italian Ministry of Health as a Scientific Institute for Research, Hospitalization and Healthcare (IRCCS). The institute promotes, coordinates and manages academic trials that arise from the innovative ideas of its researchers.

In Italy, non-profit trials are managed according to the ministerial decree of December 17, 2004. This legislation stipulates the general requirements and conditions for the execution of pharmacological clinical trials, in particular those on the improvement of clinical practice, as an integral part of healthcare. The legislation focuses on clinical trials whose promoter is a no profit organization and as such cannot be a marketing authorization holder. The ownership of the data pertaining to the trial, its execution and the results obtained belong to the promoter and the clinical trial cannot be profit-based or used for the industrial development of a drug. If these conditions are met, drug costs are paid by the National Health Service, Ethical Committee and Competent Authority fees are waived, and there are economic (i.e. minimum insurance requirements) and organizational benefits.

During the COVID-19 pandemic, clinical trial participants were in self-isolation/quarantine, with limited access to public institutes because of the risk of the spread of infection. Medical staff involved in clinical studies were also engaged in critical tasks related to the containment of the virus. Within this context, IRST, as a promoter of no profit studies, adapted its activities and procedures in accordance with European and national guidelines in an effort to maintain a high standard of clinical trials. Exceptional measures were implemented and trials were amended.

A search carried out in PubMed (https://pubmed.ncbi.nlm.nih.gov) in September 2020 using the keywords “COVID-19”, “Promoter”, “No profit”, “Management”, “Clinical trials”, “pandemic” and “guidelines”, without limits in terms of language or type of publication, revealed very little published data regarding the actions implemented by no profit clinical trial promoters during the pandemic. This prompted us to present our own experience of the measures adopted by the institute, some of which could also be used outside of a pandemic context. Our aim was also to provide useful information and strategies for other academic promoters for the management and conduction of clinical trials in a critical period such as that of the COVID-19 pandemic.

## Main text

A PubMed search using specific keywords (“COVID-19”, “Promoter”, “No profit”, “Management”, “Clinical trials”, “pandemic” and “guidelines”) identified only two methodological articles dealing with the impact of the COVID-19 pandemic on clinical trial management. Wang et al. [2] analyzed 49 clinical trial protocols of traditional Chinese medicine (TCM) for the prevention and treatment of COVID-19 to improve the quality of research design. The protocols covered the entire process of disease prevention, treatment and rehabilitation. However, the study raised numerous concerns about the unclear definition of patient characteristics, the lack of clarity of research objectives and intervention processes, and the choice of inappropriate endpoints in the protocols evaluated. Cheng et al. [3] described novel strategies for obtaining informed consent such as using video and other remote technologies in randomized clinical trials during the COVID-19 pandemic. The authors concluded that data entry should be simplified, prioritizing information of the greatest relevance.

Given the lack of published data on the management of clinical trials during the pandemic, we decided to analyze the guidelines of the Italian regulatory authorities and to describe the actions taken by our institute in an effort to facilitate the work of other academic promoters.

### IRST promoter institute of clinical trials

IRST has a Biostatistics Unit (UBSC) that manages independent studies and clinical trials sponsored by pharmaceutical companies. The Coordinating Center (CC) for IRST-promoted trials, a subunit of the UBSC, has been active since 2012. At the start of the pandemic, the CC was managing around 20 interventional studies on drugs, medical devices and radiotherapy, 13 of which (mainly phase II trials) were actively recruiting. Three of these were national multicenter trials involving 50 centers, with a total of 576 patients enrolled in active studies on March 11, 2020 (start of national lockdown), many of whom were undergoing treatment (Supplementary Table S1).

### New clinical trials and amendment submissions during the pandemic

During this period, we used an accelerated procedure to submit a new clinical trial on the use of hydroxychloroquine in patients with COVID-19 (IRST100.47 PROTECT, Eudract no. 2020-001501-24) for AIFA evaluation. Preliminary authorization was granted by AIFA’s Technical Scientific Committee (CTS), after which the study documents were assessed by AIFA’s Clinical Trials Office and a National Ethical Committee specialized in infectious diseases. Submission was handled through The National Observatory on Clinical Trials of Medicines (Osservatorio Nazionale sulla Sperimentazione Clinica dei Medicinali - OsSC) platform. We requested a pre-evaluation by the CTS on March 31, 2020 and received a suspended judgement after only 2 days (April 2, 2020). We submitted a response on April 7, 2020 and received a (final) favorable opinion on April 10, 2020. The National Ethical Committee’s favorable opinion and AIFA authorization arrived on April 20, 2020 and April 28, 2020, respectively. It took a total of 28 days for the study to be authorized compared to the 60 or more days normally required for clinical studies.

In accordance with AIFA guidelines indicating the need to reduce the number of patient accesses to trial centers, IRST successfully submitted a notified amendment via the OsSC platform and was able to authorize the participating centers of the IRST Seneca trial (IRST 100.22, Eudract no. 2016-000767-17) to supply patients with a quantity of oral drugs sufficient for more than a single therapy cycle (> 4 weeks) of “CAPTEM treatment” (one of the two arms of the trial). In an effort to facilitate patients who lived far away from the experimental centers, IRST also authorized “pre-cycle” blood tests to be performed in public medical centers near patients’ homes, with laboratory results sent to the principal investigators of participating sites. Special procedures were implemented to manage and document drug supply and accountability because of the increased amount of drugs supplied to patients. When necessary, telephone and/or video calls were used to provide detailed instructions to patients, and all communications were documented.

For the IRST 174.19 KENDO study (Eudract no. 2016-004107-31), the institute submitted a substantial amendment to notify the authorities of the temporary transfer of the Oncology Day Hospital of the Guastalla participating center to San Sebastiano Hospital in Correggio. Guastalla Hospital was re-purposed to receive the growing number of patients with COVID-19. Study staff were transferred to the new facility but blood tests continued to be performed in the Clinical Analysis Laboratory of Guastalla Hospital. The transfer to Correggio Hospital was implemented after a careful assessment of the risk of COVID-19 infection for the patients.

The above notified amendments will require formal revocation when the emergency has finished.

### Monitoring of participating centers of IRST clinical trials

IRST, as a promoter, contacted the participating centers by e-mail and telephone to evaluate the needs of each site, supervise the actions taken by investigators during the COVID-19 emergency, and give advice on how to handle specific situations. We monitored all centers to evaluate screening and recruitment trends and compliance with trial procedures. On the basis of the AIFA press release describing the possibility of modifying the management of active trials (including temporary protocol changes) to limit the risk of coronavirus infection, IRST carried out a risk assessment for each active clinical trial, introducing specific measures to reduce the number of patient accesses to hospital, continue with essential procedures, postpone or assess by telephone/teleconference (TC) all non-essential procedures, and avoid on-site visits. This remote monitoring was carried out in 50 centers participating in multicenter interventional clinical trials with drugs promoted by IRST. It revealed that patient screening was not interrupted in any site and that 16 patients were enrolled regularly by 5 centers (Table 2). When necessary, laboratory and imaging tests were carried out at COVID-19-free facilities and referrals were sent electronically to investigators who were then able to validate therapies.

**Table 2:** Comparison of data on clinical trials between the “pandemic period 2020” and the same period in 2019

	2019 (11/03–30/06)	2020 (11/03–30/06)
No. interventional trials open for enrolment	16	13
No. centers activated in multicenter trials	0	4
No. patients registered/randomized	48	53
No. on site/remote monitoring visits performed	6	11

### IRST as experimental center of clinical trials during COVID-19 pandemic

IRST, a participating center in all the trials it promoted, did not stop recruitment or treatment. IRST administration provided clear indications on the essential procedures to be maintained during the pandemic, also specifying those that could be postponed or assessed by telephone/teleconference. Patients were contacted by phone the day before a scheduled appointment to check on their health status (flu-like symptoms, fever, cough). On the day of the appointment, patients underwent triage at the hospital entrance, where nurses measured their temperature and verified the correct use of the surgical mask. Follow-up visits were not always carried out within the time-frame established by the study protocol, but rather based on the priorities defined by IRST Administration and on legal provisions.

### Actions taken by IRST as promoter to manage clinical trials

The information collected by directly contacting study sites was used by IRST’s CC to update risk assessment plans and implement suitable actions to mitigate risks. We drafted procedures and guidelines for correct trial management, in particular for telemedicine communications between clinicians and patient at home (all documented in clinical records), the shipment of laboratory blood test results, the return of unused drugs, and drug accountability. Requests for reimbursement of costs incurred by patients for laboratory exams performed at external centers were made to participating centers and will be reimbursed by the study promoter. From March 11, 2020 to June 30, 2020, all external sites involved in trials promoted by IRST were monitored remotely (Source Data Verification (SDV) was postponed), for a total of 11 monitoring visits during teleconference. Remote visits were focused on SDV as electronic patient charts were shared with the clinical monitor. It was not possible to verify source documents for only 2 remote visits. A total of 13 site initiation visits (SIV) to activate new participating centers and authorize patient recruitment were made by telephone or teleconference (Supplementary Table S2).

During the emergency, the majority of UBSC staff involved in coordinating study activities worked from home using personal computers (PCs) provided by IRST or their own PC with an adequate ADSL connection and equipped with good antivirus protection. Suitable home arrangements respecting privacy requirements were mandatory, and staff were required to undergo a brief training course on work methods, responsibilities and safety. One person manned the central office each day on a rotating basis to guarantee the continuity of necessary activities, in particular, the registration/randomization of new patients.

## Conclusions

Clinical trials must be carried out in accordance with Good Clinical Practice (GCP) guidelines, national and international laws, and standard common sense principles to ensure the rights, safety and wellbeing of participants. During the COVID-19 emergency, IRST strived to maintain the same values and standards, minimizing contact between patients and trial staff, but guaranteeing adequate supervision by the study investigator. All measures taken were well-balanced and aimed to avoid increasing the already heavy workload of personnel in participating centers [4].

EMA and AIFA produced guidelines to support clinical trial stakeholders during the pandemic, authorizing home treatment. Oral drugs were widely delivered directly to patients, but no guidance was received from AIFA nor were useful indications gained through discussion with other Italian no profit promoters with regard to the home management of infusion therapies, which are very common in oncology (national and European guidelines do not exclude the possibility of home administration). There are still no published experiences of home-based intravenous (IV) therapies in clinical trials and so patients were forced to go to hospital for IV treatments.

Very little data has been published on how the COVID-19 pandemic has impacted the management of no profit clinical trials, making it difficult to compare our experience with that of other academic promoters. The actions taken by IRST were part of the effort to adapt the activities pertaining to clinical trial management to meet regulatory authority recommendations. The final goals of clinical trials, even in an emergency period, must be to safeguard participants and guarantee the robustness and reliability of the data collected.

IRST used a home-working (HW) modality to guarantee the continuity of trial management and to maintain the regular communication between research teams in the central office and laboratory via teleconference meetings and email communications. During the emergency, continuous analysis and reporting of all protocol deviations was performed. Delays in patient screening and recruitment, drug administration, tumor restaging assessments and blood tests, these last often performed externally, had to be dealt with efficiently during the pandemic because of their potential impact on the quality of the trial results. For IRST-promoted trials, we only considered and reported in our updated procedures the protocol deviations that would have substantially impacted the primary and secondary endpoints of studies. Clinical trial risk assessment performed on a regular basis by the research team was fundamental. This epidemic could have a medium- and long-term impact on the safety of patients enrolled in clinical trials and on the quality of the data collected if adequate monitoring is not carried out. Risk mitigation plans and the implementation of specific procedures are thus needed to maintain a high level of quality in our trials.

Understanding the repercussions of the pandemic on the incidence, mortality and quality of life of cancer patients is a priority for national healthcare services worldwide. The effects of delays in diagnosis, postponed local intervention (surgery and radiotherapy) and systemic treatment, and delays in evaluating response to treatment and adverse events will have to be closely monitored. Data collected during this period will only be able to be used for publication after their robustness and reliability have been confirmed and even more caution will be needed when interpreting results for the purpose of registering new drugs.

Some of the implementations introduced by IRST have proven highly effective and suitable for use outside of the epidemic, e.g. home-working (HC), which was formally not a widespread practice in healthcare. Our experience has shown that it is possible to transfer many activities to HW, providing that there is close collaboration with colleagues remaining in the work place. Working from home is organized on the basis of phases, cycles and the achievement of objectives, established in a contract between the employer and the employee. It offers more flexible working hours, allowing employees to complete their work in a way that is most efficient for them and thus facilitating productivity. Travelling time to the workplace, with the associated stress and cost, is also eliminated, optimizing productivity. In addition, this work modality can save money for employers in terms of resources not used in the work place (equipment, office, electricity, heating, etc.). However, some employees see a downside to working from home as they feel there is no longer a clear boundary between work and private life. They may also feel isolated, cut off from their colleagues, and experience a sense of uncertainty about the quality of their work and their productivity. Overall, this new work modality requires adequate organization, a good team spirit, and the constant exchange of information and management of shared documents.

The CC staff of the UBSC underwent specific online training and held regular online meetings about the correct management of clinical trials during the pandemic to remain up-to-date and be able to compare the experience of our institute with that of other Italian stakeholders. However, the emergency highlighted that the infrastructures and technologies needed for clinical trial management must be accompanied by close collaboration between promoters and local study staff. In the future, it will be essential to have electronic medical records, possibly homogeneous at the regional/national level, and shared guidelines for a correct identification and management of risks, in particular for the protection of the rights and privacy of subjects enrolled in clinical trials.

Although clinical trial study procedures are known to be extremely stringent and structured, our experience during the pandemic suggests that some could be reviewed to increase remote promoter supervision. For example, centralized monitoring tools could be implemented to guarantee data quality and facilitate cross-checking of electronic Case Report Forms (eCRFs), with a special focus on serious adverse events (SAEs), adverse events and variables involved in primary objectives. Alternative methods of informed consent collection and trial management should introduced (on the basis of current legislation on personal data protection) to meet the needs of participants, who would obviously benefit from a simplification of trial procedures. Closer and more efficient cooperation should be pursued between the promoter and staff of participating centers through the use of clear and complete reports, check-lists and dedicated forms to facilitate study management. The ability of the promoter team to handle remote data will also help to reduce the number of activities that need to be carried out on-site, thus streamlining the duties of local staff and optimizing workloads.

## Supplementary Information

**Additional file 1: Supplementary Table S1.** Extraction from clinical trial database.

**Additional file 2: Supplementary Table S2.** Monitoring visits and Site Initiation Visits (SIV) during the “pandemic period 2020” (11/03–30/06) and the same period in 2019.

## Data Availability

The datasets generated and/or analyzed during the current study are available from the corresponding author upon reasonable request.

## Abbreviations

ADSL: Asymmetric Digital Subscriber Line
AIFA: Agenzia Italiana del Farmaco
COVID-19: COronaVIrus Disease 19
eCRF: Electronic Case Report Form
EMA: European Medicines Agency
EU/EEA: European Union/European Economic Agreement
GCP: Good Clinical Practice
SAE: Serious Adverse Event
SDV: Source Data Verification
SIV: Site Initiation Visits
HW: Home-Working
TC: Teleconference
UBSC: Unit of Biostatistics and Clinical Trials
