# Supplementary material for: Clinical research activities during COVID-19: the point of view of a promoter of academic clinical trials
Source: BMC Med Res Methodol. 2021 Apr 30;21:91. doi: 10.1186/s12874-021-01291-0 (PMC8086972; doi:10.1186/s12874-021-01291-0)
Supplement: Supplementary file 1 — Additional file 1: Supplementary Table S1. Extraction from clinical trial database. [file 12874_2021_1291_MOESM1_ESM.pdf]

Supplementary Table S1. Extraction from clinical trial database

INTERVENTIONAL TRIALS
PROMOTED BY IRST IRCCS

with ACTIVE ENROLLMENT in the period 11/03/2020-30/06/2020
with ACTIVE ENROLLMENT in the period 11/03/19-30/06/19
with patients in treatment/follow-up at 11/03/2020

**IRST100.11 LUNET – EudraCT n°2013-003165-34** **Enrollment closed on 20/03/2019**
IRCCS IRST
**IRST100.17 LUCAS – EudraCT n°2014-003067-38** **Enrollment closed on 28/08/2019**
IRCCS IRST
**IRST100.22 SENECA – EudraCT n°2016-000767-17**
AOU Careggi
AOU - Ospedali Riuniti Umberto I – G.M. Lancisi – G. Salesi - ANCONA
AOU Pisana
AOU Policlinico di Bari
AOU POLICLINICO MODENA
AOU San Luigi Gonzaga
ASST Spedali Civili di Brescia
Azienda Ospedaliera Universitaria di Parma
Azienda Ospedaliera Universitaria Policlinico "Paolo Giaccone"
Az.osp.universitaria integrata Verona-Borgo Roma
BARI IRCCS GPII
Castellana Grotte
Faenza
IRCCS - C.R.O. Centro di Riferimento Oncologico di Aviano
IRCCS IRST
Istituto Nazionale Tumori Milano
Istituto Oncologico Veneto
Milano - IEO
Ospedale di Bolzano
Ospedale di Feltre

Ospedale S. Orsola - Malpighi
Ospedale Vito Fazzi Lecce
Università Campus Bio-Medico Roma
**IRST 100.26 LUTHREE – EudraCT n°2015-004727-31**
IRCCS IRST
**IRST100.39 MESOVAX – EudraCT n°2018-000500-42**
IRCCS IRST
**IRST100.42 RAC-AD – EudraCT n°2019-000793-27**
IRCCS IRST
**IRST153.04 COREVAX-1 – EudraCT n°2015-000894-11**
IRCCS IRST
**IRST154.01 COLORE – EudraCT n°2013-000770-30 Enrollment closed on 25/05/2017**
IRCCS IRST
Ravenna
**IRST163.01 MESORT (interventional with RT)**
IRCCS IRST
**IRST172.02 ABSIDE – EudraCT n°2012-001410-41**
IRCCS IRST
**IRST172.03 IL2HD – EudraCT n°2012-001786-32**
IRCCS IRST
**IRST172.04 ACDC – EudraCT n°2014-005123-27 Enrollment and Study closed on 25/11/2019**
IRCCS IRST
**IRST174.09 met-HEReMYTA – EudraCT n°2014-002602-20 Enrollment closed on 23/05/2018**
Ferrara
Imola
IRCCS IRST
Modena
Parma
Piacenza
Rimini-Cattolica
**IRST174.19 KENDO – EudraCT n°2016-004107-31**

Ancona
Bari (CLOSED)
Bologna
Carpi
Cremona
Ferrara
Guastalla
IRCCS IRST
Legnago
Macerata
Novara
Parma
Piacenza
Ravenna
Reggio Emilia
Rimini
Sondrio
**IRST185.03 LUPSMA – EudraCT n°2016-002732-32**
IRCCS IRST
**IRST185.04 RAPSON- EudraCT n°2016-004452-29**
Aviano
Bolzano
IRCCS IRST
Lecce
Napoli (CLOSED)
Negrar
Orbassano
Parma
Pisa
Reggio Emilia
Rionero in Vulture

Trento
**IRST185.06 THRIP (interventional with RT) Enrollment temporary suspended on 23/03/2020**
IRCCS IRST
**IRST186.02 OLAPARIB – eudraCT n°2014-005095-28 Enrollment closed on 18/02/2019**
IRCCS IRST
Napoli
**IRST191.02 HBO-RT (interventional with DM + RT)**
IRCCS IRST
**IRST198.01 HIFU-BONE (interventional with DM) Enrollment closed on 05/12/2018**
IRCCS IRST

****

Study Name:	IRST100.11 LUNET	
Protocol ID:	LUNET	
Date:	2020-Oct-04	
Subjects:	68	
Study Event Definitions	1	
Study Event Definition 1	RANDOMIZATION	E1
CRF1	RANDOMIZATION [LUNET] - LUNET 1.0	C1
Study Subject ID	Protocol ID	RNDpLUNET007_E1_C1
100.11_001	LUNET - MELD_100.11	25/02/2014
100.11_003	LUNET - MELD_100.11	12/05/2014
100.11_004	LUNET - MELD_100.11	12/05/2014
100.11_007	LUNET - MELD_100.11	10/06/2014
100.11_005	LUNET - MELD_100.11	10/06/2014
100.11_006	LUNET - MELD_100.11	10/06/2014
100.11_008	LUNET - MELD_100.11	10/06/2014
100.11_009	LUNET - MELD_100.11	25/07/2014
100.11_010	LUNET - MELD_100.11	05/08/2014
100.11_011	LUNET - MELD_100.11	08/09/2014
100.11_012	LUNET - MELD_100.11	23/09/2014
100.11_013	LUNET - MELD_100.11	06/11/2014
100.11_014	LUNET - MELD_100.11	20/11/2014
100.11_015	LUNET - MELD_100.11	02/12/2014
100.11_016	LUNET - MELD_100.11	12/01/2015
100.11_017	LUNET - MELD_100.11	21/01/2015
100.11_018	LUNET - MELD_100.11	02/02/2015
100.11_019	LUNET - MELD_100.11	02/02/2015

100.11_020	LUNET - MELD_100.11	27/02/2015
100.11_021	LUNET - MELD_100.11	20/04/2015
100.11_022	LUNET - MELD_100.11	21/04/2015
100.11_023	LUNET - MELD_100.11	08/05/2015
100.11_024	LUNET - MELD_100.11	31/07/2015
100.11_025	LUNET - MELD_100.11	31/07/2015
100.11_026	LUNET - MELD_100.11	13/08/2015
100.11_027	LUNET - MELD_100.11	16/09/2015
100.11_028	LUNET - MELD_100.11	25/09/2015
100.11_029	LUNET - MELD_100.11	28/10/2015
100.11_030	LUNET - MELD_100.11	09/11/2015
100.11_031	LUNET - MELD_100.11	16/11/2015
100.11_032	LUNET - MELD_100.11	24/12/2015
100.11_033	LUNET - MELD_100.11	29/12/2015
100.11_034	LUNET - MELD_100.11	15/01/2016
100.11_035	LUNET - MELD_100.11	17/02/2016
100.11_036	LUNET - MELD_100.11	01/03/2016
100.11_038	LUNET - MELD_100.11	18/03/2016
100.11_039	LUNET - MELD_100.11	12/04/2016
100.11_040	LUNET - MELD_100.11	19/04/2016
100.11_041	LUNET - MELD_100.11	19/04/2016
100.11_042	LUNET - MELD_100.11	26/05/2016
100.11_043	LUNET - MELD_100.11	30/05/2016
100.11_044	LUNET - MELD_100.11	23/06/2016
100.11_045	LUNET - MELD_100.11	23/06/2016
100.11_046	LUNET - MELD_100.11	20/09/2016
100.11_047	LUNET - MELD_100.11	18/01/2017
100.11_048	LUNET - MELD_100.11	24/01/2017
100.11_049	LUNET - MELD_100.11	02/02/2017
100.11_050	LUNET - MELD_100.11	08/02/2017
100.11_051	LUNET - MELD_100.11	01/03/2017
100.11_052	LUNET - MELD_100.11	14/03/2017

100.11_053	LUNET - MELD_100.11	12/04/2017
100.11_054	LUNET - MELD_100.11	18/05/2017
100.11_055	LUNET - MELD_100.11	24/05/2017
100.11_056	LUNET - MELD_100.11	30/08/2017
100.11_057	LUNET - MELD_100.11	24/11/2017
100.11_058	LUNET - MELD_100.11	24/11/2017
100.11_059	LUNET - MELD_100.11	14/02/2018
100.11_060	LUNET - MELD_100.11	08/03/2018
100.11_061	LUNET - MELD_100.11	19/04/2018
100.11_062	LUNET - MELD_100.11	31/05/2018
100.11_063	LUNET - MELD_100.11	01/06/2018
100.11_064	LUNET - MELD_100.11	13/06/2018
100.11_065	LUNET - MELD_100.11	10/07/2018
100.11_066	LUNET - MELD_100.11	12/10/2018
100.11_067	LUNET - MELD_100.11	07/11/2018
100.11_068	LUNET - MELD_100.11	15/01/2019
100.11_069	LUNET - MELD_100.11	12/02/2019
100.11_070	LUNET - MELD_100.11	20/03/2019

Total patients enrolled at 11/03/2020: 68

Total patients enrolled 11/03/2019-30/06/2019: 1

Total patients enrolled 11/03/2020-30/06/2020: 0

****

Study Name:	IRST100.17 LUCAS
Protocol ID:	LUCAS
Date:	2020-Oct-04
Subjects:	33
Study Event Definitions	1
Study Event Definition 1	Randomization

CRF1 RANDOMIZATION [LUCAS] - LUCAS 1.0

Study Subject ID	Protocol ID	RNDpPATIENTINFO_010_E1_C1
100.17_001	LUCAS - MELD_100.17	11/11/2016
100.17_003	LUCAS - MELD_100.17	22/12/2016
100.17_004	LUCAS - MELD_100.17	17/01/2017
100.17_005	LUCAS - MELD_100.17	17/01/2017
100.17_006	LUCAS - MELD_100.17	18/01/2017
100.17_007	LUCAS - MELD_100.17	26/01/2017
100.17_009	LUCAS - MELD_100.17	31/01/2017
100.17_008	LUCAS - MELD_100.17	31/01/2017
100.17_011	LUCAS - MELD_100.17	12/04/2017
100.17_012	LUCAS - MELD_100.17	10/05/2017
100.17_013	LUCAS - MELD_100.17	24/05/2017
100.17_014	LUCAS - MELD_100.17	14/06/2017
100.17_015	LUCAS - MELD_100.17	14/06/2017
100.17_016	LUCAS - MELD_100.17	29/06/2017
100.17_017	LUCAS -	24/08/2017

	MELD_100.17	
	LUCAS -	
100.17_018	MELD_100.17	16/11/2017
	LUCAS -	
100.17_019	MELD_100.17	07/03/2018
	LUCAS -	
100.17_020	MELD_100.17	19/03/2018
	LUCAS -	
100.17_021	MELD_100.17	29/03/2018
	LUCAS -	
100.17_022	MELD_100.17	13/04/2018
	LUCAS -	
100.17_023	MELD_100.17	27/04/2018
	LUCAS -	
100.17_024	MELD_100.17	31/05/2018
	LUCAS -	
100.17_025	MELD_100.17	16/08/2018
	LUCAS -	
100.17_026	MELD_100.17	28/09/2018
	LUCAS -	
100.17_027	MELD_100.17	08/10/2018
	LUCAS -	
100.17_028	MELD_100.17	28/12/2018
	LUCAS -	
100.17_029	MELD_100.17	15/01/2019
	LUCAS -	
100.17_030	MELD_100.17	07/02/2019
	LUCAS -	
100.17_031	MELD_100.17	19/02/2019
	LUCAS -	
100.17_032	MELD_100.17	20/03/2019
100.17_033	LUCAS -	19/04/2019

	MELD_100.17	
	LUCAS -	
100.17_034	MELD_100.17	08/08/2019
	LUCAS -	
100.17_035	MELD_100.17	28/08/2019

Total patients enrolled at 11/03/2020: 33

Total patients enrolled 11/03/2019-30/06/2019: 3

Total patients enrolled 11/03/2020-30/06/2020: 0

****

Study Name: IRST100.22 SENECA

Protocol ID: SENECA

Date: 2020-Aug-31

Subjects: 21

Study Event Definitions 1

Study Event Definition 2 Randomization E2

CRF12 RANDOMIZATION [SENECA] - SENECA 1.0 C12

Study Subject ID	Protocol ID	RNDrRND06_E2_C12
100.22_001	SENECA - MELD_100.22	06/03/2017
100.22_002	SENECA - MELD_100.22	06/03/2017
100.22_004	SENECA - MELD_100.22	22/05/2017
100.22_003	SENECA - MILA_100.22	22/05/2017
100.22_005	SENECA - MELD_100.22	05/07/2017
100.22_006	SENECA - MILA_100.22	05/10/2017
100.22_008	SENECA - BOLZ_100.22	13/11/2017
100.22_009	SENECA - PARM_100.22	23/11/2017
100.22_011	SENECA - LECC_100.22	02/02/2018
100.22_012	SENECA - ANCO_100.22	09/02/2018

100.22_014	SENECA - FAEN_100.22	15/02/2018	
100.22_015	SENECA - LECC_100.22	30/03/2018	
100.22_016	SENECA - MODE_100.22	05/04/2018	
100.22_018	SENECA - MELD_100.22	14/05/2018	
100.22_019	SENECA - CGRO_100.22	14/05/2018	
100.22_020	SENECA - PADO_100.22	08/06/2018	
100.22_021	SENECA - PADO_100.22	12/06/2018	
100.22_022	SENECA - LECC_100.22	02/07/2018	
100.22_023	SENECA - MELD_100.22	04/07/2018	
100.22_024	SENECA - FAEN_100.22	16/07/2018	
100.22_025	SENECA - PISA_100.22	19/07/2018	
100.22_026	SENECA - PADO_100.22	24/08/2018	
100.22_027	SENECA - MILA_100.22	06/11/2018	
100.22_028	SENECA - ROMA_100.22	05/12/2018	
100.22_029	SENECA - MELD_100.22	27/12/2018	
100.22_030	SENECA - MELD_100.22	27/02/2019	
100.22_031	SENECA - MELD_100.22	09/04/2019	
100.22_046	SENECA - MIEO_100.22	1	16/03/2020
100.22_047	SENECA - PADO_100.22	25/05/2020	
100.22_051	SENECA - MELD_100.22	12/06/2020	
100.22_052	SENECA - MIEO_100.22	1	18/06/2020

Total patients enrolled at 11/03/2020: 28

Total patients enrolled 11/03/2019-30/06/2019: 1

Total patients enrolled 11/03/2020-30/06/2020: 4

****

Study Name:	IRST100.26 LUTHREE
Protocol ID:	LUTHREE
Date:	2020-Oct-04
Subjects:	370
Study Event Definitions	1

Study Event Definition 1	Randomization	E1
CRF1	RANDOMIZATION [LUTHREE] - LUTHREE 1.0	C1

Study Subject ID	Protocol ID	RNDpPATINFO009_E1_C1
100.26_001	LUTHREE - MELD_100.26	26/05/2016
100.26_002	LUTHREE - MELD_100.26	26/05/2016
100.26_003	LUTHREE - MELD_100.26	26/05/2016
100.26_004	LUTHREE - MELD_100.26	01/06/2016
100.26_005	LUTHREE - MELD_100.26	06/09/2016
100.26_006	LUTHREE - MELD_100.26	07/09/2016
100.26_007	LUTHREE - MELD_100.26	07/09/2016
100.26_008	LUTHREE - MELD_100.26	14/09/2016
100.26_009	LUTHREE - MELD_100.26	20/09/2016
100.26_010	LUTHREE - MELD_100.26	21/09/2016
100.26_011	LUTHREE - MELD_100.26	21/09/2016
100.26_012	LUTHREE - MELD_100.26	03/10/2016
100.26_013	LUTHREE - MELD_100.26	05/10/2016
100.26_014	LUTHREE - MELD_100.26	05/10/2016
100.26_016	LUTHREE - MELD_100.26	12/10/2016
100.26_015	LUTHREE - MELD_100.26	12/10/2016
100.26_018	LUTHREE - MELD_100.26	13/10/2016
100.26_017	LUTHREE - MELD_100.26	13/10/2016
100.26_019	LUTHREE - MELD_100.26	26/10/2016
100.26_020	LUTHREE - MELD_100.26	26/10/2016
100.26_024	LUTHREE - MELD_100.26	03/11/2016
100.26_023	LUTHREE - MELD_100.26	03/11/2016
100.26_022	LUTHREE - MELD_100.26	03/11/2016
100.26_021	LUTHREE - MELD_100.26	03/11/2016
100.26_025	LUTHREE - MELD_100.26	11/11/2016

100.26_026	LUTHREE - MELD_100.26	03/01/2017
100.26_027	LUTHREE - MELD_100.26	05/01/2017
100.26_029	LUTHREE - MELD_100.26	10/01/2017
100.26_028	LUTHREE - MELD_100.26	10/01/2017
100.26_030	LUTHREE - MELD_100.26	10/01/2017
100.26_031	LUTHREE - MELD_100.26	10/01/2017
100.26_032	LUTHREE - MELD_100.26	10/01/2017
100.26_033	LUTHREE - MELD_100.26	13/01/2017
100.26_036	LUTHREE - MELD_100.26	24/01/2017
100.26_039	LUTHREE - MELD_100.26	24/01/2017
100.26_038	LUTHREE - MELD_100.26	24/01/2017
100.26_034	LUTHREE - MELD_100.26	24/01/2017
100.26_037	LUTHREE - MELD_100.26	24/01/2017
100.26_035	LUTHREE - MELD_100.26	24/01/2017
100.26_040	LUTHREE - MELD_100.26	26/01/2017
100.26_041	LUTHREE - MELD_100.26	26/01/2017
100.26_042	LUTHREE - MELD_100.26	31/01/2017
100.26_043	LUTHREE - MELD_100.26	06/02/2017
100.26_044	LUTHREE - MELD_100.26	08/02/2017
100.26_045	LUTHREE - MELD_100.26	17/02/2017
100.26_046	LUTHREE - MELD_100.26	17/02/2017
100.26_048	LUTHREE - MELD_100.26	24/02/2017
100.26_049	LUTHREE - MELD_100.26	24/02/2017
100.26_047	LUTHREE - MELD_100.26	24/02/2017
100.26_050	LUTHREE - MELD_100.26	01/03/2017
100.26_051	LUTHREE - MELD_100.26	15/03/2017
100.26_052	LUTHREE - MELD_100.26	15/03/2017
100.26_055	LUTHREE - MELD_100.26	31/03/2017
100.26_053	LUTHREE - MELD_100.26	31/03/2017
100.26_054	LUTHREE - MELD_100.26	31/03/2017
100.26_056	LUTHREE - MELD_100.26	12/04/2017
100.26_058	LUTHREE - MELD_100.26	27/04/2017

100.26_057	LUTHREE - MELD_100.26	27/04/2017
100.26_059	LUTHREE - MELD_100.26	09/05/2017
100.26_060	LUTHREE - MELD_100.26	15/05/2017
100.26_061	LUTHREE - MELD_100.26	15/05/2017
100.26_062	LUTHREE - MELD_100.26	15/05/2017
100.26_063	LUTHREE - MELD_100.26	19/05/2017
100.26_064	LUTHREE - MELD_100.26	19/05/2017
100.26_065	LUTHREE - MELD_100.26	19/05/2017
100.26_066	LUTHREE - MELD_100.26	24/05/2017
100.26_067	LUTHREE - MELD_100.26	09/06/2017
100.26_068	LUTHREE - MELD_100.26	09/06/2017
100.26_069	LUTHREE - MELD_100.26	09/06/2017
100.26_070	LUTHREE - MELD_100.26	14/06/2017
100.26_071	LUTHREE - MELD_100.26	14/06/2017
100.26_072	LUTHREE - MELD_100.26	14/06/2017
100.26_073	LUTHREE - MELD_100.26	29/06/2017
100.26_074	LUTHREE - MELD_100.26	29/06/2017
100.26_075	LUTHREE - MELD_100.26	06/07/2017
100.26_076	LUTHREE - MELD_100.26	21/07/2017
100.26_077	LUTHREE - MELD_100.26	21/07/2017
100.26_078	LUTHREE - MELD_100.26	31/07/2017
100.26_079	LUTHREE - MELD_100.26	02/08/2017
100.26_080	LUTHREE - MELD_100.26	24/08/2017
100.26_081	LUTHREE - MELD_100.26	24/08/2017
100.26_082	LUTHREE - MELD_100.26	24/08/2017
100.26_083	LUTHREE - MELD_100.26	28/08/2017
100.26_084	LUTHREE - MELD_100.26	30/08/2017
100.26_085	LUTHREE - MELD_100.26	06/09/2017
100.26_086	LUTHREE - MELD_100.26	08/09/2017
100.26_087	LUTHREE - MELD_100.26	08/09/2017
100.26_088	LUTHREE - MELD_100.26	20/09/2017
100.26_089	LUTHREE - MELD_100.26	20/09/2017

100.26_090	LUTHREE - MELD_100.26	20/09/2017
100.26_091	LUTHREE - MELD_100.26	02/10/2017
100.26_092	LUTHREE - MELD_100.26	02/10/2017
100.26_093	LUTHREE - MELD_100.26	02/10/2017
100.26_094	LUTHREE - MELD_100.26	02/10/2017
100.26_095	LUTHREE - MELD_100.26	02/10/2017
100.26_096	LUTHREE - MELD_100.26	02/10/2017
100.26_097	LUTHREE - MELD_100.26	13/10/2017
100.26_098	LUTHREE - MELD_100.26	13/10/2017
100.26_099	LUTHREE - MELD_100.26	13/10/2017
100.26_100	LUTHREE - MELD_100.26	13/10/2017
100.26_101	LUTHREE - MELD_100.26	13/10/2017
100.26_102	LUTHREE - MELD_100.26	20/10/2017
100.26_103	LUTHREE - MELD_100.26	24/10/2017
100.26_104	LUTHREE - MELD_100.26	24/10/2017
100.26_105	LUTHREE - MELD_100.26	26/10/2017
100.26_106	LUTHREE - MELD_100.26	26/10/2017
100.26_107	LUTHREE - MELD_100.26	26/10/2017
100.26_108	LUTHREE - MELD_100.26	03/11/2017
100.26_109	LUTHREE - MELD_100.26	06/11/2017
100.26_110	LUTHREE - MELD_100.26	08/11/2017
100.26_111	LUTHREE - MELD_100.26	10/11/2017
100.26_112	LUTHREE - MELD_100.26	13/11/2017
100.26_113	LUTHREE - MELD_100.26	13/11/2017
100.26_114	LUTHREE - MELD_100.26	13/11/2017
100.26_115	LUTHREE - MELD_100.26	05/12/2017
100.26_116	LUTHREE - MELD_100.26	18/12/2017
100.26_117	LUTHREE - MELD_100.26	20/12/2017
100.26_118	LUTHREE - MELD_100.26	21/12/2017
100.26_119	LUTHREE - MELD_100.26	28/12/2017
100.26_120	LUTHREE - MELD_100.26	03/01/2018
100.26_121	LUTHREE - MELD_100.26	04/01/2018

100.26_122	LUTHREE - MELD_100.26	10/01/2018
100.26_123	LUTHREE - MELD_100.26	16/01/2018
100.26_124	LUTHREE - MELD_100.26	23/01/2018
100.26_125	LUTHREE - MELD_100.26	01/02/2018
100.26_126	LUTHREE - MELD_100.26	01/02/2018
100.26_127	LUTHREE - MELD_100.26	01/02/2018
100.26_128	LUTHREE - MELD_100.26	01/02/2018
100.26_129	LUTHREE - MELD_100.26	07/02/2018
100.26_130	LUTHREE - MELD_100.26	07/02/2018
100.26_131	LUTHREE - MELD_100.26	14/02/2018
100.26_132	LUTHREE - MELD_100.26	14/02/2018
100.26_133	LUTHREE - MELD_100.26	14/02/2018
100.26_134	LUTHREE - MELD_100.26	07/03/2018
100.26_135	LUTHREE - MELD_100.26	07/03/2018
100.26_136	LUTHREE - MELD_100.26	07/03/2018
100.26_137	LUTHREE - MELD_100.26	07/03/2018
100.26_138	LUTHREE - MELD_100.26	07/03/2018
100.26_139	LUTHREE - MELD_100.26	07/03/2018
100.26_140	LUTHREE - MELD_100.26	16/03/2018
100.26_141	LUTHREE - MELD_100.26	19/03/2018
100.26_142	LUTHREE - MELD_100.26	19/03/2018
100.26_143	LUTHREE - MELD_100.26	19/03/2018
100.26_144	LUTHREE - MELD_100.26	23/03/2018
100.26_145	LUTHREE - MELD_100.26	29/03/2018
100.26_146	LUTHREE - MELD_100.26	29/03/2018
100.26_147	LUTHREE - MELD_100.26	10/04/2018
100.26_148	LUTHREE - MELD_100.26	19/04/2018
100.26_149	LUTHREE - MELD_100.26	19/04/2018
100.26_150	LUTHREE - MELD_100.26	19/04/2018
100.26_151	LUTHREE - MELD_100.26	19/04/2018
100.26_152	LUTHREE - MELD_100.26	19/04/2018
100.26_153	LUTHREE - MELD_100.26	19/04/2018

100.26_154	LUTHREE - MELD_100.26	19/04/2018
100.26_155	LUTHREE - MELD_100.26	02/05/2018
100.26_156	LUTHREE - MELD_100.26	02/05/2018
100.26_157	LUTHREE - MELD_100.26	02/05/2018
100.26_158	LUTHREE - MELD_100.26	02/05/2018
100.26_159	LUTHREE - MELD_100.26	02/05/2018
100.26_160	LUTHREE - MELD_100.26	11/05/2018
100.26_161	LUTHREE - MELD_100.26	21/05/2018
100.26_163	LUTHREE - MELD_100.26	21/05/2018
100.26_164	LUTHREE - MELD_100.26	31/05/2018
100.26_165	LUTHREE - MELD_100.26	08/06/2018
100.26_166	LUTHREE - MELD_100.26	08/06/2018
100.26_167	LUTHREE - MELD_100.26	08/06/2018
100.26_168	LUTHREE - MELD_100.26	08/06/2018
100.26_169	LUTHREE - MELD_100.26	08/06/2018
100.26_170	LUTHREE - MELD_100.26	13/06/2018
100.26_171	LUTHREE - MELD_100.26	13/06/2018
100.26_172	LUTHREE - MELD_100.26	20/06/2018
100.26_173	LUTHREE - MELD_100.26	22/06/2018
100.26_174	LUTHREE - MELD_100.26	22/06/2018
100.26_175	LUTHREE - MELD_100.26	22/06/2018
100.26_176	LUTHREE - MELD_100.26	22/06/2018
100.26_177	LUTHREE - MELD_100.26	10/07/2018
100.26_178	LUTHREE - MELD_100.26	10/07/2018
100.26_179	LUTHREE - MELD_100.26	10/07/2018
100.26_180	LUTHREE - MELD_100.26	10/07/2018
100.26_181	LUTHREE - MELD_100.26	16/07/2018
100.26_182	LUTHREE - MELD_100.26	25/07/2018
100.26_183	LUTHREE - MELD_100.26	25/07/2018
100.26_184	LUTHREE - MELD_100.26	03/08/2018
100.26_185	LUTHREE - MELD_100.26	03/08/2018
100.26_186	LUTHREE - MELD_100.26	08/08/2018

100.26_187	LUTHREE - MELD_100.26	20/08/2018
100.26_188	LUTHREE - MELD_100.26	13/09/2018
100.26_189	LUTHREE - MELD_100.26	21/09/2018
100.26_190	LUTHREE - MELD_100.26	28/09/2018
100.26_191	LUTHREE - MELD_100.26	28/09/2018
100.26_192	LUTHREE - MELD_100.26	28/09/2018
100.26_193	LUTHREE - MELD_100.26	02/10/2018
100.26_194	LUTHREE - MELD_100.26	05/10/2018
100.26_195	LUTHREE - MELD_100.26	05/10/2018
100.26_196	LUTHREE - MELD_100.26	05/10/2018
100.26_197	LUTHREE - MELD_100.26	09/10/2018
100.26_198	LUTHREE - MELD_100.26	09/10/2018
100.26_199	LUTHREE - MELD_100.26	16/10/2018
100.26_200	LUTHREE - MELD_100.26	16/10/2018
100.26_201	LUTHREE - MELD_100.26	16/10/2018
100.26_202	LUTHREE - MELD_100.26	16/10/2018
100.26_203	LUTHREE - MELD_100.26	16/10/2018
100.26_204	LUTHREE - MELD_100.26	16/10/2018
100.26_205	LUTHREE - MELD_100.26	18/10/2018
100.26_206	LUTHREE - MELD_100.26	25/10/2018
100.26_207	LUTHREE - MELD_100.26	26/10/2018
100.26_208	LUTHREE - MELD_100.26	26/10/2018
100.26_209	LUTHREE - MELD_100.26	26/10/2018
100.26_210	LUTHREE - MELD_100.26	13/11/2018
100.26_211	LUTHREE - MELD_100.26	20/11/2018
100.26_212	LUTHREE - MELD_100.26	26/11/2018
100.26_213	LUTHREE - MELD_100.26	28/11/2018
100.26_214	LUTHREE - MELD_100.26	29/11/2018
100.26_215	LUTHREE - MELD_100.26	04/12/2018
100.26_216	LUTHREE - MELD_100.26	05/12/2018
100.26_217	LUTHREE - MELD_100.26	12/12/2018
100.26_218	LUTHREE - MELD_100.26	12/12/2018

100.26_219	LUTHREE - MELD_100.26	12/12/2018
100.26_220	LUTHREE - MELD_100.26	27/12/2018
100.26_221	LUTHREE - MELD_100.26	27/12/2018
100.26_222	LUTHREE - MELD_100.26	08/01/2019
100.26_223	LUTHREE - MELD_100.26	08/01/2019
100.26_224	LUTHREE - MELD_100.26	15/01/2019
100.26_225	LUTHREE - MELD_100.26	15/01/2019
100.26_226	LUTHREE - MELD_100.26	16/01/2019
100.26_227	LUTHREE - MELD_100.26	22/01/2019
100.26_228	LUTHREE - MELD_100.26	22/01/2019
100.26_229	LUTHREE - MELD_100.26	22/01/2019
100.26_230	LUTHREE - MELD_100.26	22/01/2019
100.26_231	LUTHREE - MELD_100.26	25/01/2019
100.26_232	LUTHREE - MELD_100.26	01/02/2019
100.26_233	LUTHREE - MELD_100.26	01/02/2019
100.26_234	LUTHREE - MELD_100.26	01/02/2019
100.26_235	LUTHREE - MELD_100.26	01/02/2019
100.26_236	LUTHREE - MELD_100.26	01/02/2019
100.26_237	LUTHREE - MELD_100.26	07/02/2019
100.26_238	LUTHREE - MELD_100.26	15/02/2019
100.26_239	LUTHREE - MELD_100.26	15/02/2019
100.26_240	LUTHREE - MELD_100.26	15/02/2019
100.26_241	LUTHREE - MELD_100.26	15/02/2019
100.26_242	LUTHREE - MELD_100.26	15/02/2019
100.26_243	LUTHREE - MELD_100.26	22/02/2019
100.26_244	LUTHREE - MELD_100.26	22/02/2019
100.26_245	LUTHREE - MELD_100.26	25/02/2019
100.26_246	LUTHREE - MELD_100.26	27/02/2019
100.26_247	LUTHREE - MELD_100.26	27/02/2019
100.26_248	LUTHREE - MELD_100.26	27/02/2019
100.26_249	LUTHREE - MELD_100.26	28/02/2019
100.26_250	LUTHREE - MELD_100.26	06/03/2019

100.26_251	LUTHREE - MELD_100.26	08/03/2019
100.26_252	LUTHREE - MELD_100.26	08/03/2019
100.26_253	LUTHREE - MELD_100.26	11/03/2019
100.26_254	LUTHREE - MELD_100.26	11/03/2019
100.26_255	LUTHREE - MELD_100.26	20/03/2019
100.26_256	LUTHREE - MELD_100.26	29/03/2019
100.26_257	LUTHREE - MELD_100.26	03/04/2019
100.26_258	LUTHREE - MELD_100.26	04/04/2019
100.26_259	LUTHREE - MELD_100.26	19/04/2019
100.26_260	LUTHREE - MELD_100.26	23/04/2019
100.26_261	LUTHREE - MELD_100.26	23/04/2019
100.26_262	LUTHREE - MELD_100.26	29/04/2019
100.26_263	LUTHREE - MELD_100.26	29/04/2019
100.26_264	LUTHREE - MELD_100.26	03/05/2019
100.26_265	LUTHREE - MELD_100.26	14/05/2019
100.26_266	LUTHREE - MELD_100.26	16/05/2019
100.26_267	LUTHREE - MELD_100.26	21/05/2019
100.26_268	LUTHREE - MELD_100.26	21/05/2019
100.26_269	LUTHREE - MELD_100.26	21/05/2019
100.26_270	LUTHREE - MELD_100.26	21/05/2019
100.26_271	LUTHREE - MELD_100.26	29/05/2019
100.26_272	LUTHREE - MELD_100.26	29/05/2019
100.26_273	LUTHREE - MELD_100.26	30/05/2019
100.26_274	LUTHREE - MELD_100.26	31/05/2019
100.26_275	LUTHREE - MELD_100.26	06/06/2019
100.26_276	LUTHREE - MELD_100.26	14/06/2019
100.26_277	LUTHREE - MELD_100.26	28/06/2019
100.26_278	LUTHREE - MELD_100.26	03/07/2019
100.26_279	LUTHREE - MELD_100.26	03/07/2019
100.26_280	LUTHREE - MELD_100.26	10/07/2019
100.26_281	LUTHREE - MELD_100.26	17/07/2019
100.26_282	LUTHREE - MELD_100.26	21/08/2019

100.26_283	LUTHREE - MELD_100.26	21/08/2019
100.26_284	LUTHREE - MELD_100.26	22/08/2019
100.26_285	LUTHREE - MELD_100.26	28/08/2019
100.26_286	LUTHREE - MELD_100.26	28/08/2019
100.26_287	LUTHREE - MELD_100.26	29/08/2019
100.26_288	LUTHREE - MELD_100.26	06/09/2019
100.26_289	LUTHREE - MELD_100.26	09/09/2019
100.26_290	LUTHREE - MELD_100.26	09/09/2019
100.26_291	LUTHREE - MELD_100.26	13/09/2019
100.26_292	LUTHREE - MELD_100.26	13/09/2019
100.26_293	LUTHREE - MELD_100.26	13/09/2019
100.26_294	LUTHREE - MELD_100.26	17/09/2019
100.26_295	LUTHREE - MELD_100.26	17/09/2019
100.26_296	LUTHREE - MELD_100.26	20/09/2019
100.26_297	LUTHREE - MELD_100.26	24/09/2019
100.26_298	LUTHREE - MELD_100.26	02/10/2019
100.26_299	LUTHREE - MELD_100.26	15/10/2019
100.26_300	LUTHREE - MELD_100.26	15/10/2019
100.26_301	LUTHREE - MELD_100.26	15/10/2019
100.26_302	LUTHREE - MELD_100.26	15/10/2019
100.26_303	LUTHREE - MELD_100.26	18/10/2019
100.26_304	LUTHREE - MELD_100.26	21/10/2019
100.26_305	LUTHREE - MELD_100.26	21/10/2019
100.26_306	LUTHREE - MELD_100.26	24/10/2019
100.26_307	LUTHREE - MELD_100.26	29/10/2019
100.26_308	LUTHREE - MELD_100.26	06/11/2019
100.26_309	LUTHREE - MELD_100.26	07/11/2019
100.26_310	LUTHREE - MELD_100.26	11/11/2019
100.26_311	LUTHREE - MELD_100.26	14/11/2019
100.26_312	LUTHREE - MELD_100.26	14/11/2019
100.26_313	LUTHREE - MELD_100.26	21/11/2019
100.26_314	LUTHREE - MELD_100.26	21/11/2019

100.26_315	LUTHREE - MELD_100.26	27/11/2019
100.26_316	LUTHREE - MELD_100.26	27/11/2019
100.26_317	LUTHREE - MELD_100.26	28/11/2019
100.26_318	LUTHREE - MELD_100.26	05/12/2019
100.26_319	LUTHREE - MELD_100.26	05/12/2019
100.26_320	LUTHREE - MELD_100.26	05/12/2019
100.26_321	LUTHREE - MELD_100.26	12/12/2019
100.26_322	LUTHREE - MELD_100.26	12/12/2019
100.26_323	LUTHREE - MELD_100.26	13/12/2019
100.26_324	LUTHREE - MELD_100.26	15/01/2020
100.26_325	LUTHREE - MELD_100.26	22/01/2020
100.26_326	LUTHREE - MELD_100.26	22/01/2020
100.26_327	LUTHREE - MELD_100.26	24/01/2020
100.26_328	LUTHREE - MELD_100.26	29/01/2020
100.26_329	LUTHREE - MELD_100.26	30/01/2020
100.26_330	LUTHREE - MELD_100.26	30/01/2020
100.26_331	LUTHREE - MELD_100.26	06/02/2020
100.26_332	LUTHREE - MELD_100.26	06/02/2020
100.26_333	LUTHREE - MELD_100.26	06/02/2020
100.26_334	LUTHREE - MELD_100.26	06/02/2020
100.26_335	LUTHREE - MELD_100.26	17/02/2020
100.26_336	LUTHREE - MELD_100.26	19/02/2020
100.26_337	LUTHREE - MELD_100.26	20/02/2020
100.26_338	LUTHREE - MELD_100.26	20/02/2020
100.26_339	LUTHREE - MELD_100.26	21/02/2020
100.26_340	LUTHREE - MELD_100.26	21/02/2020
100.26_341	LUTHREE - MELD_100.26	26/02/2020
100.26_342	LUTHREE - MELD_100.26	04/03/2020
100.26_343	LUTHREE - MELD_100.26	04/03/2020
100.26_344	LUTHREE - MELD_100.26	04/03/2020
100.26_345	LUTHREE - MELD_100.26	06/03/2020
100.26_346	LUTHREE - MELD_100.26	09/03/2020

100.26_347	LUTHREE - MELD_100.26	11/03/2020
100.26_348	LUTHREE - MELD_100.26	11/03/2020
100.26_349	LUTHREE - MELD_100.26	11/03/2020
100.26_350	LUTHREE - MELD_100.26	11/03/2020
100.26_351	LUTHREE - MELD_100.26	25/03/2020
100.26_352	LUTHREE - MELD_100.26	25/03/2020
100.26_353	LUTHREE - MELD_100.26	01/04/2020
100.26_354	LUTHREE - MELD_100.26	08/04/2020
100.26_355	LUTHREE - MELD_100.26	15/04/2020
100.26_356	LUTHREE - MELD_100.26	15/04/2020
100.26_357	LUTHREE - MELD_100.26	22/04/2020
100.26_358	LUTHREE - MELD_100.26	29/04/2020
100.26_359	LUTHREE - MELD_100.26	30/04/2020
100.26_360	LUTHREE - MELD_100.26	06/05/2020
100.26_361	LUTHREE - MELD_100.26	20/05/2020
100.26_362	LUTHREE - MELD_100.26	25/05/2020
100.26_363	LUTHREE - MELD_100.26	27/05/2020
100.26_364	LUTHREE - MELD_100.26	29/05/2020
100.26_365	LUTHREE - MELD_100.26	29/05/2020
100.26_366	LUTHREE - MELD_100.26	03/06/2020
100.26_367	LUTHREE - MELD_100.26	04/06/2020
100.26_368	LUTHREE - MELD_100.26	04/06/2020
100.26_369	LUTHREE - MELD_100.26	12/06/2020
100.26_370	LUTHREE - MELD_100.26	18/06/2020
100.26_371	LUTHREE - MELD_100.26	18/06/2020

Total patients enrolled at 11/03/2020: 345

Total patients enrolled 11/03/2019-30/06/2019: 25

Total patients enrolled 11/03/2020-30/06/2020: 25

****

Study Name: IRST100.39 MESOVAX

Protocol ID: MESOVAX

Date: 2020-Oct-02

Subjects: 3

Study Event Definitions 1

Study Event Definition 2 Eligibility E2

CRF27 ELIGIBILITY CONFIRMATION [MESOVAX] - MESOVAX 0.1 C27

Study Subject ID	Protocol ID	ELGeDATEENROLL_E2_C27
100.39_002	MESOVAX - MELD_100.39	19/12/2019
100.39_004	MESOVAX - MELD_100.39	12/12/2019
100.39_005	MESOVAX - MELD_100.39	26/03/2020

Total patients enrolled at 11/03/2020: 3

Total patients enrolled 11/03/2019-30/06/2019: 0

Total patients enrolled 11/03/2020-30/06/2020: 1

****

Study Name: IRST100.42 RAC-AD

Protocol ID: RAC-AD

Date: 2020-Oct-02

Subjects: 0

Study Event Definitions 0

Study Subject ID Protocol ID

Total patients enrolled at 11/03/2020: 0

Total patients enrolled 11/03/2019-30/06/2019: 0

Total patients enrolled 11/03/2020-30/06/2020: 0

****

Study Name: IRST153.04 COREVAX-1

Protocol ID: COREVAX-1

Date: 2020-Oct-02

Subjects: 0

Study Event Definitions 0

Study Subject ID	Protocol ID	RGSpPATIENTINF5_E1_C2
153.04_001	COREVAX-1 - MELD_153.04	02/12/2016
153.04_002	COREVAX-1 - MELD_153.04	07/04/2017
153.04_003	COREVAX-1 - MELD_153.04	30/05/2017
153.04_004	COREVAX-1 - MELD_153.04	19/06/2017
153.04_005	COREVAX-1 - MELD_153.04	03/07/2017
153.04_006	COREVAX-1 - MELD_153.04	16/11/2017
153.04_007	COREVAX-1 - MELD_153.04	20/02/2018
153.04_008	COREVAX-1 - MELD_153.04	08/05/2018
153.04_009	COREVAX-1 - MELD_153.04	01/04/2019
153.04_010	COREVAX-1 - MELD_153.04	07/01/2020
153.04_011	COREVAX-1 - MELD_153.04	09/03/2020

Total patients enrolled at 11/03/2020: 11

Total patients enrolled 11/03/2019-30/06/2019: 1

Total patients enrolled 11/03/2020-30/06/2020: 0

****

Study Name:	IRST163.01 MESORT	
Protocol ID:	MESORT	
Date:	2020-Oct-04	
Subjects:		22
Study Event Definitions		1

Study Event Definition 2	REGISTRATION CONFIRMATION	E2
	REGISTRATION CONFIRMATION	
CRF13	[MESORT] - MESORT 0.1	C13

Study Subject ID	Protocol ID

RGCelELIGIBILITY006_E2_C13

163.01_002	MESORT - MELD_163.01	30/08/2017
163.01_001	MESORT - MELD_163.01	05/09/2017
163.01_003	MESORT - MELD_163.01	08/09/2017
163.01_004	MESORT - MELD_163.01	08/09/2017
163.01_005	MESORT - MELD_163.01	09/10/2017
163.01_006	MESORT - MELD_163.01	20/10/2017
163.01_007	MESORT - MELD_163.01	13/11/2017
163.01_008	MESORT - MELD_163.01	05/12/2017
163.01_009	MESORT - MELD_163.01	23/01/2018
163.01_010	MESORT - MELD_163.01	02/02/2018
163.01_011	MESORT - MELD_163.01	17/04/2018
163.01_012	MESORT - MELD_163.01	20/06/2018
163.01_013	MESORT - MELD_163.01	30/07/2018
163.01_014	MESORT - MELD_163.01	21/09/2018
163.01_016	MESORT - MELD_163.01	09/10/2018
163.01_017	MESORT - MELD_163.01	17/10/2018
163.01_018	MESORT - MELD_163.01	08/01/2019
163.01_019	MESORT - MELD_163.01	13/02/2019
163.01_020	MESORT - MELD_163.01	27/02/2019
163.01_021	MESORT - MELD_163.01	12/06/2019
163.01_022	MESORT - MELD_163.01	19/06/2019
163.01_023	MESORT - MELD_163.01	20/05/2020

Total patients enrolled at 11/03/2020: 21

Total patients enrolled 11/03/2019-30/06/2019: 2

Total patients enrolled 11/03/2020-30/06/2020: 1

****

Study Name: IRST172.02 ABSIDE

Protocol ID: ABSIDE

Date: 2020-Oct-04

Subjects: 26

Study Event Definitions 1

Study Event Definition 1 Randomization

CRF1 RANDOMIZATION - ABSIDE 1.0

Study Subject ID	Protocol ID	RANDpABSIDE006_E1_C1
172.02_001	ABSIDE - MELD_172.02	08/10/2013
172.02_002	ABSIDE - MELD_172.02	21/10/2013
172.02_003	ABSIDE - MELD_172.02	19/02/2014
172.02_004	ABSIDE - MELD_172.02	17/03/2014
172.02_010	ABSIDE - MELD_172.02	29/04/2014
172.02_005	ABSIDE - MELD_172.02	05/05/2014
172.02_007	ABSIDE - MELD_172.02	18/12/2014
172.02_011	ABSIDE - MELD_172.02	11/06/2015
172.02_012	ABSIDE - MELD_172.02	27/01/2016
172.02_013	ABSIDE - MELD_172.02	23/02/2016
172.02_014	ABSIDE - MELD_172.02	29/04/2016
172.02_015	ABSIDE -	05/05/2016

	MELD_172.02	
172.02_017	ABSIDE - MELD_172.02	13/10/2016
172.02_018	ABSIDE - MELD_172.02	18/10/2016
172.02_019	ABSIDE - MELD_172.02	24/10/2016
172.02_020	ABSIDE - MELD_172.02	21/12/2016
172.02_021	ABSIDE - MELD_172.02	18/01/2017
172.02_022	ABSIDE - MELD_172.02	21/03/2017
172.02_023	ABSIDE - MELD_172.02	04/12/2017
172.02_024	ABSIDE - MELD_172.02	28/12/2017
172.02_026	ABSIDE - MELD_172.02	09/04/2018
172.02_027	ABSIDE - MELD_172.02	23/04/2019
172.02_028	ABSIDE - MELD_172.02	26/04/2019
172.02_029	ABSIDE - MELD_172.02	17/01/2020
172.02_030	ABSIDE - MELD_172.02	15/06/2020
172.02_031	ABSIDE - MELD_172.02	16/06/2020

Total patients enrolled at 11/03/2020: 24

Total patients enrolled 11/03/2019-30/06/2019: 2

Total patients enrolled 11/03/2020-30/06/2020: 2

****

Study Name: IRST172.03 IL2HD

Protocol ID: IL2HD

Date: 2020-Oct-05

Subjects: 28

Center	ID Center	ID patient	Registration Date
IRST IRCCS	1	1	09-lug-12
IRST IRCCS	1	2	03-ott-12
IRST IRCCS	1	3	18-ott-12
IRST IRCCS	1	4	10-dic-12
IRST IRCCS	1	5	12-feb-13
IRST IRCCS	1	6	10-apr-13
IRST IRCCS	1	7	27-nov-13
IRST IRCCS	1	8	06-mar-14
IRST IRCCS	1	9	06-giu-14
IRST IRCCS	1	10	01-set-14
IRST IRCCS	1	11	03-mar-15

IRST IRCCS	1	12	07-apr-15
IRST IRCCS	1	13	12-mag-15
IRST IRCCS	1	14	19-giu-15
IRST IRCCS	1	15	04-ago-15
IRST IRCCS	1	16	11-ago-15
IRST IRCCS	1	17	04-dic-15
IRST IRCCS	1	18	12-feb-16
IRST IRCCS	1	19	09-mag-16
IRST IRCCS	1	20	20-set-16
IRST IRCCS	1	21	11-apr-17
IRST IRCCS	1	22	01-ago-17
IRST IRCCS	1	23	10-ago-18
IRST IRCCS	1	24	06-mag-19
IRST IRCCS	1	25	13-mag-19
IRST IRCCS	1	26	23-set-19

IRST IRCCS	1	27	18-mag-20
IRST IRCCS	1	28	11-giu-20

Total patients enrolled at 11/03/2020: 26

Total patients enrolled 11/03/2019-30/06/2019: 2

Total patients enrolled 11/03/2020-30/06/2020: 2

****

Study Name: IRST172.04 ACDC

Protocol ID: ACDC adjuvant trial

Date: 2020-Oct-05

Subjects: 18

Study Event Definitions 1

Study Event Definition 1 Randomization

RANDOMIZATION [ACDC] - ACDC

CRF1 1.0

Study Subject ID	Protocol ID	RNDpDATERANDOMIZATION_E1_C1
172.04_001	ACDC adjuvant trial - MELD_172.04	17/09/2015
172.04_002	ACDC adjuvant trial - MELD_172.04	29/01/2016
172.04_003	ACDC adjuvant trial - MELD_172.04	08/04/2016
172.04_004	ACDC adjuvant trial - MELD_172.04	22/04/2016
172.04_005	ACDC adjuvant trial -	15/07/2016

	MELD_172.04	
172.04_006	ACDC adjuvant trial - MELD_172.04	27/10/2016
172.04_007	ACDC adjuvant trial - MELD_172.04	13/01/2017
172.04_008	ACDC adjuvant trial - MELD_172.04	13/04/2017
172.04_009	ACDC adjuvant trial - MELD_172.04	16/08/2017
172.04_010	ACDC adjuvant trial - MELD_172.04	15/09/2017
172.04_011	ACDC adjuvant trial - MELD_172.04	27/09/2017
172.04_012	ACDC adjuvant trial - MELD_172.04	20/12/2017
172.04_013	ACDC adjuvant trial - MELD_172.04	14/02/2018
172.04_014	ACDC adjuvant trial - MELD_172.04	20/03/2018
172.04_015	ACDC adjuvant trial - MELD_172.04	21/03/2018
172.04_016	ACDC adjuvant trial - MELD_172.04	08/06/2018
172.04_017	ACDC adjuvant trial - MELD_172.04	14/06/2018
172.04_018	ACDC adjuvant trial - MELD_172.04	27/11/2018

Total patients enrolled at 11/03/2020: 18

Total patients enrolled 11/03/2019-30/06/2019: 0

Total patients enrolled 11/03/2020-30/06/2020: 0

****

Study Name:	IRST174.09 met-HEReMYTA	
Protocol ID:	met-HEReMYTA	
Date:	2020-Oct-05	
Subjects:	49	
Study Event Definitions	1	
Study Event Definition 1	Registration	E1
CRF1	REGISTRATION [met-HEReMYTA] - 1.0 met-HEReMYTA	C1

Study Subject ID	Protocol ID	RGSpMETHEREMYTA009_E1_C1
174.09_001	met-HEReMYTA - IRST_174.09	17/12/2014
174.09_002	met-HEReMYTA - IRST_174.09	13/02/2015
174.09_003	met-HEReMYTA - RIMI_174.09	02/04/2015
174.09_004	met-HEReMYTA - IRST_174.09	01/09/2015
174.09_005	met-HEReMYTA - IRST_174.09	24/09/2015
174.09_006	met-HEReMYTA - IRST_174.09	09/10/2015
174.09_007	met-HEReMYTA - IRST_174.09	09/11/2015
174.09_008	met-HEReMYTA - MODE_174.09	30/11/2015
174.09_009	met-HEReMYTA - IRST_174.09	03/12/2015
174.09_010	met-HEReMYTA - IRST_174.09	04/01/2016
174.09_011	met-HEReMYTA - IRST_174.09	04/02/2016
174.09_012	met-HEReMYTA - IRST_174.09	11/02/2016
174.09_013	met-HEReMYTA - MODE_174.09	19/02/2016
174.09_014	met-HEReMYTA - IRST_174.09	03/03/2016
174.09_016	met-HEReMYTA - IRST_174.09	07/03/2016
174.09_015	met-HEReMYTA - IRST_174.09	07/03/2016
174.09_017	met-HEReMYTA - IRST_174.09	09/03/2016
174.09_018	met-HEReMYTA - MODE_174.09	08/04/2016
174.09_019	met-HEReMYTA - MODE_174.09	22/04/2016
174.09_020	met-HEReMYTA - MODE_174.09	25/05/2016

174.09_021	met-HEReMYTA - MODE_174.09	03/06/2016
174.09_022	met-HEReMYTA - IRST_174.09	10/06/2016
174.09_023	met-HEReMYTA - IRST_174.09	01/08/2016
174.09_024	met-HEReMYTA - RIMI_174.09	15/12/2016
174.09_025	met-HEReMYTA - RIMI_174.09	10/02/2017
174.09_026	met-HEReMYTA - MODE_174.09	27/04/2017
174.09_027	met-HEReMYTA - RIMI_174.09	12/05/2017
174.09_028	met-HEReMYTA - IRST_174.09	25/05/2017
174.09_029	met-HEReMYTA - MODE_174.09	29/05/2017
174.09_030	met-HEReMYTA - IRST_174.09	04/07/2017
174.09_031	met-HEReMYTA - IRST_174.09	31/08/2017
174.09_032	met-HEReMYTA - IRST_174.09	05/09/2017
174.09_033	met-HEReMYTA - RIMI_174.09	11/09/2017
174.09_034	met-HEReMYTA - IMOL_174.09	05/10/2017
174.09_035	met-HEReMYTA - RIMI_174.09	06/10/2017
174.09_036	met-HEReMYTA - IRST_174.09	11/10/2017
174.09_037	met-HEReMYTA - MODE_174.09	08/11/2017
174.09_038	met-HEReMYTA - IMOL_174.09	17/11/2017
174.09_039	met-HEReMYTA - IRST_174.09	07/02/2018
174.09_040	met-HEReMYTA - IRST_174.09	28/02/2018
174.09_041	met-HEReMYTA - IMOL_174.09	22/03/2018
174.09_042	met-HEReMYTA - RIMI_174.09	04/04/2018
174.09_043	met-HEReMYTA - RIMI_174.09	17/04/2018
174.09_044	met-HEReMYTA - IRST_174.09	20/04/2018
174.09_045	met-HEReMYTA - IRST_174.09	24/04/2018
174.09_046	met-HEReMYTA - IMOL_174.09	30/04/2018
174.09_047	met-HEReMYTA - PIAC_174.09	14/05/2018
174.09_048	met-HEReMYTA - MODE_174.09	22/05/2018
174.09_049	met-HEReMYTA - IMOL_174.09	23/05/2018

Total patients enrolled at 11/03/2020: 49

Total patients enrolled 11/03/2019-30/06/2019: 0

Total patients enrolled 11/03/2020-30/06/2020: 0

****

Study Name:	IRST174.19 KENDO	
Protocol ID:	CHENDO	
Date:	2020-Oct-05	
Subjects:		41
Study Event Definitions		1
Study Event Definition 2	Randomization	E2
CRF20	RANDOMIZATION [CHENDO] - CHENDO 2.0	C20
CRF21	RANDOMIZATION [CHENDO] - CHENDO 1.0	C21

Study Subject ID	Protocol ID	RNDrRANDOMIZATIONDATE_E2_C20
174.19_001	CHENDO - MELD_174.19	07/08/2017
174.19_002	CHENDO - RAFALU_174.19	04/09/2017
174.19_003	CHENDO - CARP_174.19	04/10/2017
174.19_004	CHENDO - MELD_174.19	17/10/2017
174.19_005	CHENDO - MELD_174.19	28/12/2017
174.19_006	CHENDO - MELD_174.19	08/01/2018
174.19_007	CHENDO - MELD_174.19	11/01/2018
174.19_008	CHENDO - RAFALU_174.19	24/01/2018
174.19_009	CHENDO - MELD_174.19	06/02/2018
174.19_010	CHENDO - MELD_174.19	19/02/2018
174.19_011	CHENDO - RAFALU_174.19	28/02/2018
174.19_012	CHENDO - CARP_174.19	07/03/2018
174.19_013	CHENDO - MELD_174.19	20/04/2018
174.19_014	CHENDO - RAFALU_174.19	14/05/2018

174.19_015	CHENDO - MELD_174.19	24/05/2018
174.19_016	CHENDO - MELD_174.19	14/06/2018
174.19_018	CHENDO - PIAC_174.19	19/02/2019
174.19_019	CHENDO - PIAC_174.19	21/02/2019
174.19_020	CHENDO - MELD_174.19	21/03/2019
174.19_021	CHENDO - CREM_174.19	02/05/2019
174.19_022	CHENDO - MELD_174.19	28/05/2019
174.19_023	CHENDO - MELD_174.19	19/06/2019
174.19_024	CHENDO - MELD_174.19	08/07/2019
174.19_025	CHENDO - LEGN_174.19	11/07/2019
174.19_027	CHENDO - MELD_174.19	09/09/2019
174.19_028	CHENDO - MELD_174.19	06/11/2019
174.19_029	CHENDO - MELD_174.19	27/01/2020
174.19_030	CHENDO - CARP_174.19	10/02/2020
174.19_031	CHENDO - CARP_174.19	12/02/2020
174.19_033	CHENDO - RIMI_174.19	21/02/2020
174.19_032	CHENDO - MELD_174.19	24/02/2020
174.19_034	CHENDO - MELD_174.19	05/03/2020
174.19_035	CHENDO - MELD_174.19	23/03/2020
174.19_036	CHENDO - CARP_174.19	31/03/2020
174.19_037	CHENDO - CARP_174.19	03/04/2020
174.19_038	CHENDO - MELD_174.19	05/05/2020
174.19_039	CHENDO - FERR_174.19	06/05/2020
174.19_040	CHENDO - MELD_174.19	08/05/2020
174.19_041	CHENDO - MELD_174.19	08/05/2020
174.19_043	CHENDO - MELD_174.19	01/06/2020
174.19_042	CHENDO - MELD_174.19	04/06/2020

Total patients enrolled at 11/03/2020: 32

Total patients enrolled 11/03/2019-30/06/2019: 4

Total patients enrolled 11/03/2020-30/06/2020: 9

****

Study Name: IRST 185.03 LUPSMA

Protocol ID: LUPSMA

Date: 2020-Oct-05

Subjects: 68

Study Event Definitions 1

Study Event Definition 2	Registration confirmation	E2
CRF6	REGISTRATION CONFIRMATION [LUPSMA] - LUPSMA 0.1	C6

Study Subject ID	Protocol ID	RGCelELIGIBILITY006_E2_C6
185.03_009	LUPSMA - MELD_185.03	22/06/2017
185.03_010	LUPSMA - MELD_185.03	22/06/2017
185.03_011	LUPSMA - MELD_185.03	22/06/2017
185.03_013	LUPSMA - MELD_185.03	24/08/2017
185.03_012	LUPSMA - MELD_185.03	12/09/2017
185.03_014	LUPSMA - MELD_185.03	12/09/2017
185.03_015	LUPSMA - MELD_185.03	16/10/2017
185.03_016	LUPSMA - MELD_185.03	23/10/2017
185.03_017	LUPSMA - MELD_185.03	23/10/2017
185.03_018	LUPSMA - MELD_185.03	23/10/2017
185.03_019	LUPSMA - MELD_185.03	03/11/2017
185.03_020	LUPSMA - MELD_185.03	03/11/2017
185.03_021	LUPSMA - MELD_185.03	08/11/2017
185.03_022	LUPSMA - MELD_185.03	09/11/2017
185.03_023	LUPSMA - MELD_185.03	20/11/2017
185.03_024	LUPSMA - MELD_185.03	13/12/2017
185.03_025	LUPSMA - MELD_185.03	17/01/2018

185.03_026	LUPSMA - MELD_185.03	23/02/2018
185.03_027	LUPSMA - MELD_185.03	19/03/2018
185.03_028	LUPSMA - MELD_185.03	28/03/2018
185.03_029	LUPSMA - MELD_185.03	28/03/2018
185.03_030	LUPSMA - MELD_185.03	04/04/2018
185.03_031	LUPSMA - MELD_185.03	04/04/2018
185.03_032	LUPSMA - MELD_185.03	11/05/2018
185.03_033	LUPSMA - MELD_185.03	21/05/2018
185.03_001	LUPSMA - MELD_185.03	05/06/2018
185.03_002	LUPSMA - MELD_185.03	05/06/2018
185.03_003	LUPSMA - MELD_185.03	05/06/2018
185.03_004	LUPSMA - MELD_185.03	05/06/2018
185.03_005	LUPSMA - MELD_185.03	05/06/2018
185.03_006	LUPSMA - MELD_185.03	05/06/2018
185.03_007	LUPSMA - MELD_185.03	05/06/2018
185.03_008	LUPSMA - MELD_185.03	05/06/2018
185.03_034	LUPSMA - MELD_185.03	29/06/2018
185.03_035	LUPSMA - MELD_185.03	03/07/2018
185.03_037	LUPSMA - MELD_185.03	06/08/2018
185.03_036	LUPSMA - MELD_185.03	27/08/2018
185.03_038	LUPSMA - MELD_185.03	05/09/2018
185.03_039	LUPSMA - MELD_185.03	25/10/2018
185.03_040	LUPSMA - MELD_185.03	22/11/2018
185.03_042	LUPSMA - MELD_185.03	22/11/2018
185.03_041	LUPSMA - MELD_185.03	26/11/2018
185.03_043	LUPSMA - MELD_185.03	27/11/2018
185.03_044	LUPSMA - MELD_185.03	11/02/2019
185.03_046	LUPSMA - MELD_185.03	20/03/2019
185.03_047	LUPSMA - MELD_185.03	29/03/2019
185.03_048	LUPSMA - MELD_185.03	03/04/2019
185.03_049	LUPSMA - MELD_185.03	10/05/2019
185.03_050	LUPSMA - MELD_185.03	21/05/2019

185.03_051	LUPSMA - MELD_185.03	22/05/2019
185.03_052	LUPSMA - MELD_185.03	02/07/2019
185.03_053	LUPSMA - MELD_185.03	03/07/2019
185.03_054	LUPSMA - MELD_185.03	24/07/2019
185.03_055	LUPSMA - MELD_185.03	09/08/2019
185.03_056	LUPSMA - MELD_185.03	23/08/2019
185.03_057	LUPSMA - MELD_185.03	27/08/2019
185.03_058	LUPSMA - MELD_185.03	08/10/2019
185.03_059	LUPSMA - MELD_185.03	04/11/2019
185.03_061	LUPSMA - MELD_185.03	11/12/2019
185.03_062	LUPSMA - MELD_185.03	17/01/2020
185.03_063	LUPSMA - MELD_185.03	06/03/2020
185.03_064	LUPSMA - MELD_185.03	06/03/2020
185.03_065	LUPSMA - MELD_185.03	16/03/2020
185.03_066	LUPSMA - MELD_185.03	25/03/2020
185.03_067	LUPSMA - MELD_185.03	21/04/2020
185.03_068	LUPSMA - MELD_185.03	19/05/2020
185.03_069	LUPSMA - MELD_185.03	05/06/2020
185.03_070	LUPSMA - MELD_185.03	23/06/2020

Total patients enrolled at 11/03/2020: 62

Total patients enrolled 11/03/2019-30/06/2019: 6

Total patients enrolled 11/03/2020-30/06/2020: 6

****

Study Name: IRST185.04 RAPSON

Protocol ID: RAPSON

Date: 2020-Oct-02

Subjects: 5

Study Event Definitions 1

Study Event Definition 2 Randomization E2

CRF24 RANDOMIZATION [RAPSON] - RAPSON 1.0 C24

Study Subject ID	Protocol ID	RNDrRANDOMIZATIONDATE_E2_C24
185.04_001	RAPSON - MELD_185.04	01/09/2017
185.04_002	RAPSON - BOLZ_185.04	17/11/2017
185.04_004	RAPSON - MELD_185.04	03/04/2018
185.04_005	RAPSON - MELD_185.04	03/04/2018
185.04_006	RAPSON - NEGR_185.04	08/05/2018
185.04_007	RAPSON - MELD_185.04	29/05/2018
185.04_008	RAPSON - PISA_185.04	20/06/2018
185.04_009	RAPSON - PARM_185.04	05/07/2018
185.04_011	RAPSON - MELD_185.04	26/02/2020
185.04_012	RAPSON - MELD_185.04	03/03/2020
185.04_013	RAPSON - MELD_185.04	14/04/2020
185.04_014	RAPSON - MELD_185.04	27/05/2020
185.04_015	RAPSON - MELD_185.04	01/06/2020

Total patients enrolled at 11/03/2020: 10

Total patients enrolled 11/03/2019-30/06/2019: 0

Total patients enrolled 11/03/2020-30/06/2020: 3

****

Study Name:	IRST185.06 THRIP	
Protocol ID:	THRIP	
Date:	2020-Oct-05	
Subjects:		2
Study Event Definitions		1

Study Event Definition 2	Registration Confirmation	E2
CRF12	REGISTRATION CONFIRMATION [THRIP] - THRIP 0.1	C12

Study Subject ID	Protocol ID	RGCelELIGIBILITY006_E2_C12
185.06_001	THRIP - MELD_185.06	30/10/2018
185.06_002	THRIP - MELD_185.06	06/09/2019

Total patients enrolled at 11/03/2020: 2

Total patients enrolled 11/03/2019-30/06/2019: 0

Total patients enrolled 11/03/2020-30/06/2020: 0

****

Study Name: IRST186.02 OLAPARIB

Protocol ID: OLAPARIB

Date: 2020-Oct-02

Subjects: 0

Study Event Definitions 0

Study Subject ID	Protocol ID	RGSpOLAPARIB010_E1_C1
186.02_01_001	OLAPARIB - MELD_186.02	11/09/2015
186.02_01_003	OLAPARIB - MELD_186.02	17/09/2015
186.02_01_002	OLAPARIB - MELD_186.02	17/09/2015
186.02_01_004	OLAPARIB - MELD_186.02	01/10/2015
186.02_02_005	OLAPARIB - NAPO_186.02	06/10/2015
186.02_01_006	OLAPARIB - MELD_186.02	08/10/2015
186.02_01_007	OLAPARIB - MELD_186.02	04/12/2015
186.02_02_008	OLAPARIB - NAPO_186.02	06/09/2016
186.02_02_009	OLAPARIB - NAPO_186.02	09/03/2017
186.02_01_010	OLAPARIB - MELD_186.02	14/02/2018
186.02_01_011	OLAPARIB - MELD_186.02	14/02/2018
186.02_01_012	OLAPARIB - MELD_186.02	09/03/2018
186.02_01_013	OLAPARIB - MELD_186.02	26/04/2018
186.02_01_014	OLAPARIB - MELD_186.02	14/05/2018
186.02_01_016	OLAPARIB - MELD_186.02	25/06/2018

186.02_01_017	OLAPARIB - MELD_186.02	20/09/2018
186.02_01_018	OLAPARIB - MELD_186.02	07/11/2018
186.02_01_019	OLAPARIB - MELD_186.02	18/02/2019

Total patients enrolled at 11/03/2020: 18

Total patients enrolled 11/03/2019-30/06/2019: 0

Total patients enrolled 11/03/2020-30/06/2020: 0

****

Study Name:	IRST191.02 HBO-RT	
Protocol ID:	HBO-RT	
Date:	2020-Oct-05	
Subjects:		13
Study Event Definitions		1
Study Event Definition 2	Registration Confirmation	E2
CRF7	REGISTRATION CONFIRMATION [HBO-RT] - HBO-RT 0.1	C7

Study Subject ID	Protocol ID	RGCelELIGIBILITY006_E2_C7
191.02_001	HBO-RT - MELD_191.02	26/04/2018
191.02_002	HBO-RT - MELD_191.02	04/05/2018
191.02_004	HBO-RT - MELD_191.02	22/05/2018
191.02_003	HBO-RT - MELD_191.02	23/05/2018
191.02_005	HBO-RT - MELD_191.02	23/05/2018
191.02_006	HBO-RT - MELD_191.02	25/06/2018
191.02_007	HBO-RT - MELD_191.02	24/07/2018
191.02_008	HBO-RT - MELD_191.02	22/08/2018
191.02_009	HBO-RT - MELD_191.02	03/09/2018
191.02_010	HBO-RT - MELD_191.02	28/09/2018

191.02_011	HBO-RT - MELD_191.02	12/04/2019
191.02_012	HBO-RT - MELD_191.02	23/08/2019
191.02_013	HBO-RT - MELD_191.02	02/10/2019

Total patients enrolled at 11/03/2020: 13

Total patients enrolled 11/03/2019-30/06/2019: 1

Total patients enrolled 11/03/2020-30/06/2020: 0

****

Study Name:	IRST198.01 HIFU-BONE	
Protocol ID:	HIFU-BONE	
Date:	2020-Oct-05	
Subjects:		12
Study Event Definitions		1
Study Event Definition 1	Registration	E1
CRF1	REGISTRATION [HIFU-BONE] - HIFU-BONE 0.1	C1

Study Subject ID	Protocol ID	RGSeELEG003_E1_C1
198.01_001	HIFU-BONE - MELD_198.01	14/09/2015
198.01_002	HIFU-BONE - MELD_198.01	22/12/2015
198.01_003	HIFU-BONE - MELD_198.01	16/01/2016
198.01_004	HIFU-BONE - MELD_198.01	27/01/2016
198.01_005	HIFU-BONE - MELD_198.01	14/03/2016
198.01_006	HIFU-BONE - MELD_198.01	30/03/2016
198.01_007	HIFU-BONE - MELD_198.01	27/06/2016
198.01_008	HIFU-BONE - MELD_198.01	12/12/2016
198.01_009	HIFU-BONE - MELD_198.01	25/08/2017
198.01_010	HIFU-BONE - MELD_198.01	26/09/2017

198.01_011	HIFU-BONE - MELD_198.01	28/03/2018
198.01_012	HIFU-BONE - MELD_198.01	24/04/2018

Total patients enrolled at 11/03/2020: 12

Total patients enrolled 11/03/2019-30/06/2019: 0

Total patients enrolled 11/03/2020-30/06/2020: 0
