# Supplementary material for: Clinical research activities during COVID-19: the point of view of a promoter of academic clinical trials
Source: BMC Med Res Methodol. 2021 Apr 30;21:91. doi: 10.1186/s12874-021-01291-0 (PMC8086972; doi:10.1186/s12874-021-01291-0)
Supplement: Supplementary file 2 — Additional file 2: Supplementary Table S2. Monitoring visits and Site Initiation Visits (SIV) during the “pandemic period 2020” (11/03–30/06) and the same period in 2019. [file 12874_2021_1291_MOESM2_ESM.pdf]

Supplementary Table S2. Monitoring visits and Site Initiation Visits (SIV) during the "pandemic period 2020" (11/03-30/06) and the same period in 2019.

Date of monitoring visit	Study
15/03/2019	IRST153.04 COREVAX
07/05/2019	IRST191.02 HBO RT
24/05/2019	IRST100.22 SENECA
27/05/2019	IRST174.09 met-Heremyta
04/06/2019	IRST185.06 THRIP
10/06/2019	IRST172.02 ABSIDE
01/04/2020	IRST100.39 MESOVAX
23/04/2020	BEATcc
04/05/2020	BEATcc
13/05/2020	IRST100.39 MESOVAX
29/05/2020	IRST174.19 KENDO
03/06/2020	IRST174.19 KENDO
09/06/2020	IRST172.02 ABSIDE
16/06/2020	IRST100.39 MESOVAX
22/06/2020	BEATcc
24/06/2020	IRST172.02 ABSIDE
26/06/2020	BEATcc

Date of SIV	Study
11/03/2020	IRST100.22 SENECA
25/05/2020	IRST185.04 RAPSON
28/05/2020	IRST185.04 RAPSON
12/06/2020	IRST185.04 RAPSON
16/04/2020	IRST185.04 RAPSON
17/06/2020	IRST185.04 RAPSON
15/06/2020	IRST185.04 RAPSON
08/04/2020	BEATcc
06/05/2020	IRST100.22 SENECA
20/05/2020	BEATcc
29/05/2020	BEATcc
26/06/2020	IRST100.22 SENECA
29/06/2020	BEATcc
